# Validating the accuracy of the Hendrich II Fall Risk Model for hospitalized patients using the ROC curve analysis

**DOI:** 10.1002/kjm2.12807

**Published:** 2024-02-16

**Authors:** Chieh‐Ying Hu, Li‐Chen Sun, Ming‐Yen Lin, Mei‐Hsing Chen, Hsin‐Tien Hsu

**Affiliations:** ^1^ Integrated Long‐Term Care Services Center, Kaohsiung Municipal Ta‐Tung Hospital Kaohsiung Medical University Kaohsiung Taiwan; ^2^ Department of Nursing, Kaohsiung Medical University Hospital Kaohsiung Medical University Kaohsiung Taiwan; ^3^ Division of Nephrology, Department of Internal Medicine, Kaohsiung Medical University Hospital Kaohsiung Medical University Kaohsiung Taiwan; ^4^ Superintendent Office, Kaohsiung Medical University Hospital Kaohsiung Medical University Kaohsiung Taiwan; ^5^ Center for Quality Management and Patient Safety, Kaohsiung Medical University Hospital Kaohsiung Medical University Kaohsiung Taiwan; ^6^ School of Nursing Kaohsiung Medical University Kaohsiung Taiwan; ^7^ Department of Medical Research, Kaohsiung Medical University Hospital Kaohsiung Medical University Kaohsiung Taiwan

**Keywords:** fall risk assessment, falls, inpatients, receiver operating characteristic (ROC), the Hendrich II Fall Risk Model (HIIFRM)

## Abstract

This retrospective study was conducted at a medical center in southern Taiwan to assess the accuracy of the Hendrich II Fall Risk Model (HIIFRM) in predicting falls. Sensitivity, specificity, accuracy, and optimal cutoff points were analyzed using receiver operating characteristic (ROC) curves. Data analysis was conducted using information from the electronic medical record and patient safety reporting systems, capturing 303 fall events and 47,146 non‐fall events. Results revealed that at the standard threshold of HIIFRM score ≥5, the median score in the fall group was significantly higher than in the non‐fall group. The top three units with HIIFRM scores exceeding 5 were the internal medicine (50.6%), surgical (26.5%), and oncology wards (14.1%), indicating a higher risk of falls in these areas. ROC analysis showed an HIIFRM sensitivity of 29.5% and specificity of 86.3%. The area under the curve (AUC) was 0.57, indicating limited discriminative ability in predicting falls. At a lower cutoff score (≥2), the AUC was 0.75 (95% confidence interval: 0.666–0.706; *p* < 0.0001), suggesting acceptable discriminative ability in predicting falls, with an additional identification of 101 fall events. This study emphasizes the importance of selecting an appropriate cutoff score when using the HIIFRM as a fall risk assessment tool. The findings have implications for fall prevention strategies and patient care in clinical settings, potentially leading to improved outcomes and patient safety.

## 1 INTRODUCTION

According to the 2021 Taiwan Patient Safety Reporting System, hospital falls ranked second in the number of reported incidents, accounting for 22.5% of the total reports. An analysis of the impact of various events on patient health revealed that falls were associated with the highest proportion of severe injuries (37.5%). This not only leads to prolonged hospitalization but also increases medical costs, causing significant effects on patients and their families. The highest occurrence of falls was in general wards, accounting for approximately 79.2% of every hundred reported cases. Being male and aged 65 years and above had the highest number of incidents (50.0% and 47.4%, respectively) (Taiwan Patient Safety Reporting System, 2021).

According to the data analysis of hospital patient fall incidents, 9031 patients were assessed as high‐risk, accounting for 74.1% of the total, before the occurrence of falls. In other words, nearly one‐fourth of hospitalized patients were not identified as high risk before the events occurred (Taiwan Patient Safety Reporting System, 2021). In clinical settings, the use of a fall risk assessment tool with high validity and reliability is essential for successfully identifying individuals who are prone to falls. Yet, nearly 30% of patients in our institution were not identified as high‐risk before the occurrence of fall events, missing the opportunity for timely implementation of preventive measures. These findings emphasize the importance of continuous improvements in fall prevention measures to ensure early identification and targeted intervention for high‐risk patients.

After conducting a literature search and evaluating the accuracy of various fall risk‐assessment tools, the Hendrich II Fall Risk Model (HIIFRM), Morse Fall Scale (MFS), The Johns Hopkins Fall Risk Assessment Tool (JHFRAT), and the St. Thomas Risk Assessment Tool in Falling Elderly Inpatients (STRATIFY) were selected for initial analysis.^1^, ^2^, ^3^, ^4^, ^5^, ^6^ Of these, the STRATIFY tool failed to predict fall risk for patients admitted to geriatric wards and those aged 75 and older, particularly those aged 75–84. The MFS and JHFRAT showed lower sensitivity and specificity than the HIIFRM. A systematic review and meta‐analysis conducted by Park (2018) supported our selection of the HIIFRM in assessing fall risk among elderly patients admitted to the medical center selected for our study.^7^ The HIIFRM demonstrated favorable accuracy, with a sensitivity of 74.9%, specificity of 73.9%, negative predictive value of 99.5%, and positive predictive value of 2%.^2^ With its cutoff point of ≥5, the HIIFRM allows for early identification and intervention for individuals at a higher risk of falling, potentially preventing falls and reducing associated injuries. Overall, the HIIFRM is known to perform reasonably well in correctly identifying fallers and non‐fallers, achieving a balance between sensitivity and specificity. Considering its reliability, ease of implementation, and ability to accurately assess fall risk in hospitalized elderly patients, the HIIFRM was selected as the preferred fall risk assessment tool for predicting high‐risk fall patients in our study.

The HIIFRM was initially introduced in 2003 by Hendrich, Bender, and Nyhuis, with a cutoff point of 5 on a scale of 0–16.^2^ It is an evidence‐based fall risk‐assessment tool commonly used in geriatric acute healthcare settings. Nurses can easily and quickly use it and complete the assessment within 60–90 s. However, in a subsequent study conducted in 2020 by Hendrich, Bufalibe, and Groves, the cutoff point was revised to 4. The revisited study revealed that by lowering the cutoff point, an additional 74 patients who would have been overlooked if the original cutoff point of 5 had been retained were identified.^3^ This finding aligns with the experience of our hospital, where patients at a high risk for falls were not detected despite the use of a fall risk‐assessment tool.

To validate the accuracy of the HIIFRM as a reliable assessment tool for identifying high‐risk fall patients, we employed a receiver operating characteristic (ROC) curve analysis. This analysis aimed to assess the tool's accuracy, thereby providing healthcare professionals with a more robust mechanism for predicting high‐risk fall patients, and establishing adjustments of clinical care protocols for implementing appropriate preventive measures to ensure patient safety. The primary objective of this study was to determine an accurate cutoff point for the HIIFRM specifically tailored to Asian healthcare systems, thereby enhancing its applicability in this context.

## 2 METHODS

### 2.1 Study design and patient recruitment

We retrospectively reviewed the medical records of all adult patients admitted to the study hospitals between January and December 2017. This study aimed to encompass a comprehensive sample of adult patients aged 18 years and above from a 1700‐bed medical center. Outpatients, emergency rooms, and pediatric patients (<18 years of age) were excluded from the analysis. The demographic characteristics of the participants (including sex, age, and hospitalization ward) were collected from hospitalization records, and data from the patient safety reporting system on inpatient falls were merged with these records. Statistical analyses were performed on the data classified into two groups: patients who experienced inpatient falls and those who did not.

From January to December 2016, 22 education and training sessions were held by the nursing staff to establish the correct use of HIIFRM before it was fully implemented in adult inpatients. Continuous quality control monitoring was used to evaluate the accuracy of the nursing staff's use of the HIIFRM in adult inpatients. The results of quality control monitoring showed that the accuracy rate of the nursing staff's use of HIIFRM was 98.6%, indicating that the use of HIIFRM by nursing staff was highly accurate. The nurses completed the HIIFRM before the end of their shift after admitting the patient.

### 2.2 The HIIFRM tool

In a study by Hendrich et al. (2003), patients and controls (355/780) were randomly enrolled and assessed for more than 600 risk factors (intrinsic/extrinsic). The instrument consists of eight variables. It includes questions regarding confusion/disorientation/impulsivity (No = 0, Yes = 4); symptomatic depression (No = 0, Yes = 2); altered elimination (No = 0, Yes = 1); dizziness/vertigo (No = 0, Yes = 1); sex (female = 0, male = 1); any administered antiepileptics (No = 0, Yes = 2); any administered benzodiazepines (No = 0, Yes = 1); and the “get up and go” test (0–4) which assesses the patient's ability to stand up from a sitting position. Each risk factor is assigned a score depending on the calculated relative risk. A total score of 5 or greater indicates a high risk of fall. This tool was developed specifically for the risk assessment of patient falls in hospitals, and the scale requires only 3–5 min to complete.^2^ In previous studies, HIIFRM was found to be applicable for hospitalized elderly individuals, with a sensitivity of 0.76 (95% confidence interval (CI): 0.68–0.83), specificity of 0.60 (95% CI: 0.57–0.62), and an ROC Area Under the Curve (AUC) of 0.75 (SE = 0.05).^1^, ^8^, ^9^

### 2.3 Statistical analysis

In the study, a fall is defined as “*an unplanned descent to the floor with or without injury to the patient*” (Taiwan Patient Safety Reporting System, 2021). Descriptive data are presented as mean, standard deviation (SD), and proportion (%). Predictive validity was estimated by measuring the sensitivity, specificity, ROC curve, and AUC.

Statistical differences between the fall and non‐fall groups were calculated using the *χ* ^2^ and two‐tailed *t*‐tests. Using the Mann–Whitney *U* test for a two‐sample *t*‐test, we examined the difference in means between the fall and non‐fall groups. Monte Carlo simulation involves assigning multiple values to uncertain variables to obtain various results and then averaging those results to obtain an estimate.^10^ Using the parameters of the HIIFRM, 47,449 Monte Carlo simulations were conducted to simulate disease attributes in different units. ROC is the most common method used to evaluate the discriminative abilities of diagnostic tools. The size of the AUC indicates the degree of desirable sensitivity and specificity. The AUC values range from 0 to 1, with a higher value indicating better discriminative ability. AUC values between 0.8 and 0.9 represent good discriminative ability, whereas those between 0.9 and 1.0 represent an excellent discriminative ability.^11^ In addition, the positive predictive value (PPV) is the ratio of the number of true high‐risk fall cases to the total number of cases predicted as high‐risk falls, whereas the negative predictive value (NPV) is the ratio of the number of true non‐high‐risk fall cases to the total number of cases predicted as non‐high‐risk falls, with accuracy being the ratio of the total number of correct predictions to the total number of cases. High PPV and NPV values close to 100 suggest that the tool is as good as the reference tool.^12^, ^13^

Youden's index is frequently employed in conjunction with ROC analysis to evaluate diagnostic tests that provide numeric results instead of dichotomous outcomes. The index is defined for every point on the ROC curve, and the maximum value of the index serves as a criterion for determining the optimal cutoff point. Using Youden's index, researchers can effectively identify the threshold that maximizes both sensitivity and specificity, aiding in the selection of an optimal cutoff point for diagnostic tests with continuous numerical outputs.^14^

All forms were processed and statistically analyzed using SPSS 20.0 software. Demographic data and fall events were analyzed using the *χ* ^2^ test. The accuracy and cutoff point calculations of HIIFRM were analyzed using ROC curves, and sensitivity, specificity, PPV, NPV, and accuracy were established.

### 2.4 Ethical considerations

The study was approved by the Institutional Review Board (IRB #KMUHIRB‐E(I)‐20230137). Regarding the confidentiality and privacy protection of the participants' data, patient medical records and safety notification system data were accessed and utilized in accordance with the hospital's administrative procedures, and following approval, the designated personnel from the information department obtained the approved data and provided it to the project leader. This was then locked, requiring a password to access it, which was retained by the project leader. Individual patient information was not collected in the research data, nor did any such information appear in the data analysis or research publications.

## 3 RESULTS

### 3.1 Characteristics of the patients observed falls

As shown in Table 1, the study included a diverse range of patients, with ages ranging from 19 to 98 years. The average age for the non‐fall group was 56.7 ± 18.2 years, while the average age for the fall group was 62.7 ± 15.9 years. The mean age of the fallers was significantly higher than that of the non‐fallers. The odds of falling were found to increase with age [odds ratio (OR), 95% CI: 1.013, 1.008–1.018]. The age distribution of the participants was as follows: 62.7% were between the ages of 19 and 64 years, 26.6% between 65 and 79 years, and 10.7% were aged 80 years and above. The male‐to‐female ratio was equal among participants. Of all fallers, it was found that the incidence of falls was significantly higher in male patients than in female patients (63.0% vs. 37.0%).

**TABLE 1:** Frequency distribution of the demographic and study variables.

Characteristics	Overall	Without fall event	Fall event	*p* value
Number of hospitalized patients, *n*	31,743	31,486	257	
Age, years, mean ± SD		56.7 ± 18.2	62.7 ± 15.9	<0.0001
Age, OR (95% CI)	1.013 (1.008–1.018)			<0.0001
19–64, *n* (%)	19,912 (62.7)	19,776 (62.8)	136 (52.9)	0.0017
65–79, *n* (%)	8438 (26.6)	8358 (26.6)	80 (31.1)	
≥80, *n* (%)	3393 (10.7)	3352 (10.7)	41 (16.0)	
Sex, *n* (%)				<0.0001
Male	15,533 (48.9)	15,371 (48.8)	162 (63.0)	
Female	16,210 (51.1)	16,115 (51.2)	95 (37.0)	
Number of total hospitalizations, *N*	47,449	47,146	303	
Type of unit				<0.001^a^
Internal medical	16,962 (35.7)	16,795 (35.6)	167 (55.1)	
Surgical	10,541 (22.2)	10,506 (22.3)	35 (11.6)	
Intensive Care	572 (1.2)	572 (1.2)	0 (0.0)	
Gynecology and Obstetrics	1935 (4.1)	1930 (4.1)	5 (1.6)	
Rehabilitation	1739 (3.7)	1721 (3.7)	18 (5.9)	
Psychiatry	412 (0.9)	396 (0.8)	16 (5.3)	
Oncology	15,163 (31.9)	15,101 (32.0)	62 (20.5)	
Other^b^	125 (0.3)	125 (0.3)	0 (0.0)	

Abbreviations: CI, confidence interval; OR, odds ratio.

^a^ Monte Carlo estimated for the exact test.

^b^ Other, includes the Respiratory Care Center (RCC), and Radiation Ward.

### 3.2 Characteristics of the patients within the HIIFRM with and without fall events

Analysis of risk factors within the HIIFRM revealed statistically significant differences between individuals who experienced falls and those who did not. Specifically, confusion/disorientation/impulsivity increased the risk of falling by 4.92 times (95% CI = [2.67, 9.08]), whereas symptomatic depression increased the risk of falling by 3.36 times (95% CI = [1.65, 6.84]). Altered elimination increased the risk of falling by 3.6 times (95% CI = [2.00, 6.47]), whereas dizziness/vertigo increased the risk of falling by 5.24 times (95% CI = [2.30, 11.93]). Being male increased the risk of falling by 2.27 times (95% CI = [1.74, 2.97]), with administered antiepileptics increasing the risk of falling by 7.87 times (95% CI = [4.94, 12.55]), and administered Benzodiazepines increasing the risk of falling by 2.54 times (95% CI = [1.30, 4.97]). Finally, the “Get‐up‐and‐Go” test in being “Unable to rise without assistance” during testing increased the risk of falling by 6.93 times (95% CI = [4.87, 9.85]). The median score HIIFRM was 2.0 with an interquartile range (IQR) of 3.0, whereas for patients who did not experience falls, the median score was 1.0 with an IQR of 2.0. The study found that the median HIIFRM score in the fall group was significantly higher than that in the non‐fall group (Mann–Whitney *U* test, *p* < 0.0001) (Table 2).

**TABLE 2:** Points of risk factors within the HIIFRM with and without fall events.

Number of person‐times, *N*	Overall	Without fall event	Fall event	Odds ratio	95% CI		*p* value
	47,449	47,146	303		Low limit	Up limit	
Confusion/disorientation/impulsivity							<0.001
None	46,215 (97.4)	45,933 (97.4)	282 (93.1)	1.00 [Reference]			
Yes	1234 (2.6)	1213 (2.6)	21 (6.9)	4.92	2.67	9.08	
Symptomatic depression							<0.001
None	46,982 (99.0)	46,688 (99.0)	294 (97.0)	1.00 [Reference]			
Yes	467 (1.0)	458 (1.0)	9 (3.0)	3.36	1.65	6.84	
Altered elimination (missing *n* = 519)							<0.001
None	46,049 (98.1)	45,762 (98.1)	287 (95.7)	1.00 [Reference]			
Yes	881 (1.9)	868 (1.9)	13 (4.3)	3.6	2.00	6.47	
Dizziness/Vertigo (missing *n* = 1823)							<0.001
None	45,395 (99.5)	45,127 (99.5)	268 (97.8)	1.00 [Reference]			
Yes	231 (0.5)	225 (0.5)	6 (2.2)	5.24	2.30	11.93	
Male							<0.001
None	23,474 (49.5)	23,376 (49.6)	98 (32.3)	1.00 [Reference]			
Yes	23,975 (50.5)	23,770 (50.4)	205 (67.7)	2.27	1.74	2.97	
Any prescribed antiepileptics							<0.001
None	46,465 (97.9)	46,185 (98.0)	280 (92.4)	1.00 [Reference]			
Yes	984 (2.1)	961 (2.0)	23 (7.6)	7.87	4.94	12.55	
Any prescribed benzodiazepines							0.0047
None	46,765 (98.6)	46,471 (98.6)	294 (97.0)	1.00 [Reference]			
Yes	684 (1.4)	675 (1.4)	9 (3.0)	2.54	1.30	4.97	
Get‐up‐and‐Go Test: “Rising from a chair”							<0.001
Ability to rise in a single movement	28,000 (68.6)	27,953 (68.9)	77 (30.3)	1.00 [Reference]			
Pushes up, successful in one attempt	7544 (18.5)	7455 (18.4)	89 (35)	4.33	3.19	5.89	
Multiple attempts but successful	2445 (6.0)	2410 (5.9)	35 (13.8)	5.27	3.53	7.88	
Unable to rise without assistance	2831 (6.9)	2778 (6.8)	53 (20.9)	6.93	4.87	9.85	
HIIFRM score, median (IQR)	1.0 (2.0)	1.0 (2.0)	2.0 (3.0)				<0.0001^a^

*Note*: Data is represented as count (percentage).

Abbreviations: CI, confidence interval; OR, odds ratio.

^a^ Mann–Whitney *U* test.

The median score of the HIIFRM was 1.0 on a 0–16 point scale. Of the total HIIFRM scores, 76.4% were below 2 and 6.0% were equal to or above 5. This table presents the corresponding scores (weights) assigned to each item within the HIIFRM tool, offering valuable insights into the prevalence of specific risk factors and their associated weights. Among the wards included in the study, the proportions of HIIFRM scores exceeding 5 were highest in the internal medicine (IM) ward (50.6%), followed by the surgical (SUR) (26.5%) and oncological (ONC) (14.1%) wards, indicating a higher prevalence of patients at risk of falling in these areas. Notably, the ward with the lowest proportion of HIIFRM scores exceeding five points was other [wards] (including RCC and the radiation ward). Among the majority of patients in IM, 51.6% were male, 8.3% often experienced dizziness/vertigo, 5.6% had altered elimination, 5.0% experienced confusion/disorientation/impulsivity (score 4), and 8.4% required assistance to stand up from a chair (score 4). Among the patients in the SUR ward, the factors prevalent were: being male, administration of antiepileptics, dizziness/vertigo, and altered elimination, accounting for 52.7%, 3.1%, 3.0%, and 1.7% of the patients, respectively. Notably, the “Get‐up‐and‐Go” test was not feasible for a significant number of patients in the ICU, with 74.9% unable to provide this information. Among these patients, excluding male members, the most frequently observed risk factors were related to confusion/disorientation/impulsivity, administration of antiepileptics, and dizziness/vertigo (5.6%, 4.6%, 3.0%), respectively (Table 3). These findings provide valuable insights into the HIIFRM assessment outcomes across different hospital units.

**TABLE 3:** Frequency of each HIIFRM fall risk item in the eight units.

Item	Item score	IM *n* = 13,938	SUR *n* = 19,495	ICU *n* = 539	G&O *n* = 3318	REH *n* = 479	PSY *n* = 417	ONC *n* = 9095	OTH *n* = 168
Confusion/disorientation/impulsivity	4	5.0%	1.6%	5.6%	0.1%	5.4%	3.1%	1.6%	1.2%
Symptomatic depression	2	1.2%	0.7%	0.9%	0.2%	3.3%	24.5%	0.4%	1.2%
Altered elimination	1	5.6%	1.7%	2.2%	1.3%	11.7%	1.2%	1.8%	1.8%
Dizziness/Vertigo	1	8.3%	3.0%	3.0%	1.1%	6.3	7.2%	2.2%	3.0%
Sex (male)	1	51.6%	52.7%	62.9%	0.0%	61.2%	42.4%	60.8%	26.8%
Antiepileptics	2	1.3%	3.1%	4.6%	0.2%	13.2%	2.4%	0.6%	1.2%
Benzodiazepines	1	1.7%	1.6%	0.7%	0.4%	2.7%	5.3%	0.9%	1.2%
Get‐up‐and‐Go test									
“Ability to rise in a single movement”	0	40.7%	64.2%	7.9%	93.6%	30.4%	73.7%	77.6%	25.0%
“Pushes up, successful in one attempt”	1	19.7%	17.0%	6.4%	4.3%	17.7%	14.9%	12.0%	9.7%
“Multiple attempts but successful”	3	7.8%	5.0%	5.0%	0.7%	8.2%	2.1%	2.3%	1.6%
“Unable to rise without assistance”	4	8.4%	4.3%	5.9%	0.5%	15.9%	4.7%	3.7%	5.6%
“Not feasible” (*n* = 6629)	‐	23.3%	9.5%	74.9%	1.0%	27.9%	4.5%	4.3%	58.1%
HIIFRM score	*N* (%)								
≥5	2843 (6.0)	50.6%	26.5%	1.5%	0.3%	6.0%	0.9%	14.1%	0.1%
4	2729 (5.8)	46.8%	34.3%	1.4%	1.0%	2.6%	0.8%	13.1%	0.1%
3	1856 (3.9)	41.1%	42.7%	1.6%	1.2%	1.9%	2.4%	9.1%	0.1%
2	4696 (9.9)	36.8%	44.6%	0.7%	0.3%	1.1%	1.7%	14.7%	0.0%
1	19,748 (41.6)	27.3%	45.0%	1.3%	1.0%	0.5%	0.7%	24.0%	0.2%
0	15,577 (32.8)	21.5%	38.7%	0.9%	19.6%	0.4%	0.7%	17.5%	0.7%

Abbreviations: G&O, gynecology and obstetrics; ICU, intensive care unit; IM, internal medicine; ONC, oncology; OTH, other; PSY, psychiatry; REH, rehabilitation; SUR, surgery.

### 3.3 Accuracy and the optimal cutoff point of HIIFRM

**FIGURE 1:**
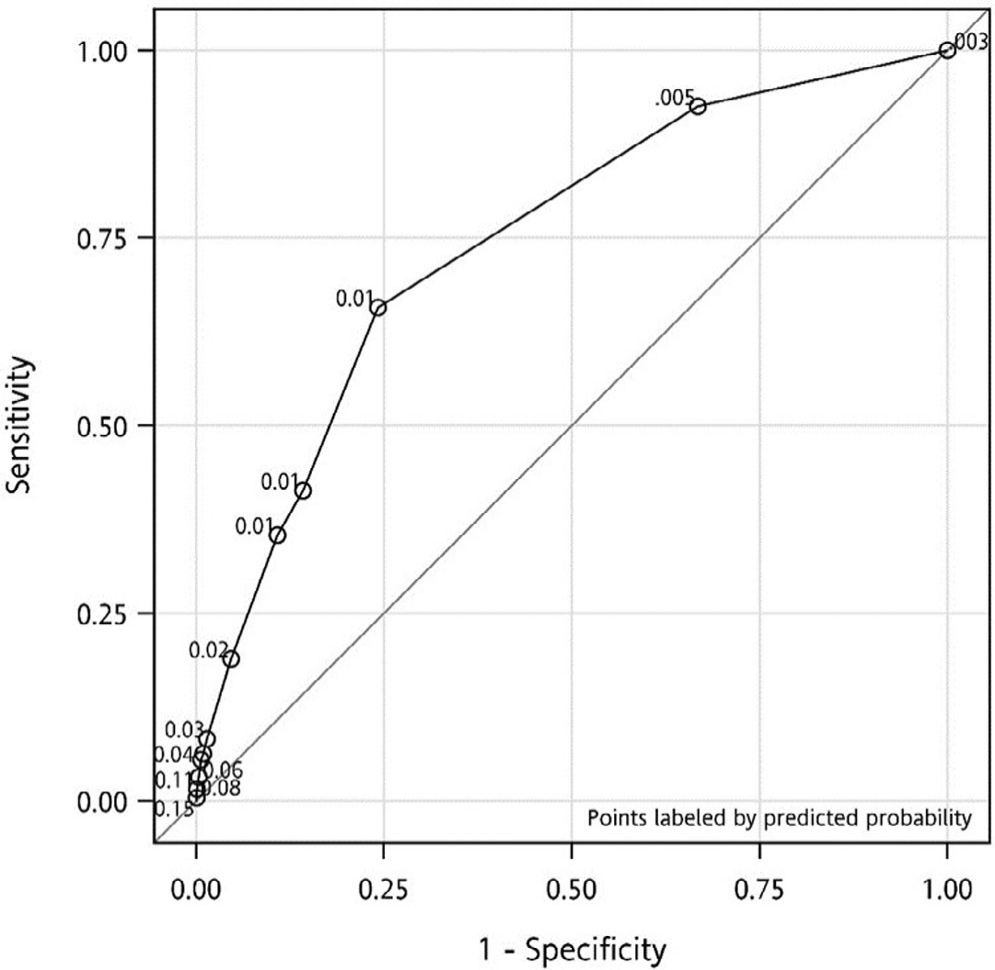
Receiver operating characteristic (ROC) curve of HIIFRM.

In this study, statistical analysis was conducted on 303 fall events and 47,146 non‐fall individuals. To ensure the reliability of any diagnostic technique, the AUC needs to be greater than 0.5 and ideally greater than 0.7 for fairness.^15^ The Youden index determined the optimal cutoff point for the HIIFRM to be 2, with corresponding sensitivity, specificity, PPV, and NPV values of 69.5%, 64.4%, 1.7%, and 99.7%, respectively (Table 4). For patients in the ICU who are comatose and unable to perform the “Get‐Up‐and‐Go” test, it is recommended to consider the test as “not applicable” and neither assign a score of 0 nor 4. However, in the 6629 cases where the “Get‐Up‐and‐Go” test could not be performed, particularly for patients in wheelchairs, based on the medical records at the time of testing, a score of 4 is assigned if the patient requires assistance from another person to stand. The ROC curve in Figure 1 shows an AUC of 0.745 (*p* < 0.0001, 95% CI: 0.666–0.706), indicating a moderate predictive capability of the scale.

**TABLE 4:** Accuracy of the HIIFRM in predicting hospitalized fall events.

Total score	SEN, %	SPE, %	PPV, %	NPV, %	Accuracy, %	Youden index	AUC
≥1	90.2	28.6	0.9	99.9	33.6	0.19	0.63
≥2	69.5	64.4	1.7	99.7	75.7	0.34	0.75
≥3	51.8	73.5	1.8	99.6	85.5	0.25	0.64
≥4	45.8	76.7	2.0	99.6	88.8	0.23	0.62
≥5	29.5	86.3	2.5	99.5	94.9	0.16	0.57
≥6	11.2	95.3	3.4	99.4	98	0.07	0.53
≥7	8.1	96.8	3.9	99.4	98.5	0.05	0.53
≥8	5.9	97.7	4.9	99.4	98.7	0.04	0.52
≥9	2.8	98.6	5.1	99.4	99	0.01	0.51

Abbreviations: AUROC, area under the receiver operating characteristic curve; NPV, negative predicted value; PPV, positive predicted value; SEN, sensitivity; SPE, specificity.

## 4 DISCUSSION

At the standard threshold of HIIFRM score ≥5, the study identified 303 falls and 47,146 non‐falls, resulting in a sensitivity of 29.5% and specificity of 86.3%. The area under the ROC curve was 0.57, suggesting that the HIIFRM may not be highly accurate in predicting falls. However, employing a lower cutoff score of ≥2 resulted in an improved sensitivity of 69.5%, and a reduced specificity of 64.4%, with a Youden index of 0.34, indicating moderate accuracy in predicting falls. The area under the ROC curve was 0.75, demonstrating better overall performance at this cutoff point. A cutoff of ≥2 would add 9281 patients to the 2843 patients with scores ≥5, yielding a higher fall predictability for a total of 12,124 at‐risk inpatients, or 25.6% of the entire sample. There were 303 fall events, and among patients with scores greater than 2, 101 had scores less than 5. This highlights the need for improvement in the patient safety culture toward “zero falls.” Additionally, factors such as confusion/disorientation/impulsivity, symptomatic depression, altered elimination, dizziness/vertigo, sex, administration of antiepileptics, administration of Benzodiazepines and the “Get‐up‐and‐Go” test showed significant differences between the fall and non‐fall groups.

Hospital falls are multifactorial in nature. Previous publications have reported the risk factors for falls, including age, sex, balance impairment, dizziness upon standing, and cognitive impairment/confusion.^16^, ^17^, ^18^ Several studies have reported an increased risk of falls with advancing age.^16^ In our study, we also observed a significant difference in the average age of the fall group compared with that of the non‐fall group, suggesting that older age was a contributing factor.^17^ Although falls can occur at any age, their frequency increases with age.^7^, ^17^ According to Shuyi et al. (2022) sex is a factor affecting the frequency of falls in older adults.^19^ Notably, the non‐fall group in this study comprised approximately equal proportions of male and female patients, although in the fall group, we observed a significantly higher risk of falls among male compared to female patients. The consideration of sex as a factor is essential in developing effective fall prevention strategies, as highlighted in this study and corroborated by others.^18^, ^19^, ^20^

In the present study, most patients had a chronic disease, particularly hypertension, coronary artery disease, depression, hypoglycemic symptoms^21^ or cancer, and were regularly taking medications for their chronic diseases.^17^ These treatments may involve the use of medications, such as antidepressants, antipsychotics, benzodiazepines, diuretics, sedatives, and chemotherapy drugs, which could exacerbate the risk of falls in adult patients with chronic illnesses, leading to muscle weakness, poor balance, impaired proprioception, impaired cognition, and functional disabilities, particularly in the three units reflecting the highest HIIFRM fall scores.^22^, ^23^ During pregnancy, women experience significant physiological, anatomical and metabolic changes distinct from acute or older individuals. As pregnancy progresses, factors such as pregnancy‐related hypotension, frequent urination, and shifts in the body's center of gravity can increase the risk of falling. However, it should be noted that previous studies using the HIIFRM did not include pregnant women as participants.^24^, ^25^, ^26^ Resultantly, whether the HIIFRM is effective for assessing the risk of falls in pregnant women requires further research and confirmation.

Regarding the “‘Get‐Up‐and‐Go” test, patients who required multiple attempts to rise were almost three times more likely to fall than those who were able to rise in a single movement or those who pushed themselves up successfully in one attempt (OR = 5.27). Similarly, patients who were unable to rise without assistance during the test were almost seven times more likely to fall than those who were able to rise in a single movement or those who successfully pushed themselves up in a single attempt (OR = 6.93). While these values are slightly higher than those of some studies,^2^, ^8^ the comprehensive results of the study indicate that attributes such as “multiple attempts but successful” and “unable to rise without assistance” during the “Get‐Up‐and‐Go” test have the most significant impact on the occurrence of falls. In the ICU, risk factors for falls such as confusion, sedation, and prolonged immobility are related to the acute care needs of patients.^27^ If a patient is comatose, they should be closely monitored and scored completely once an assessment is completed. If the patient is not comatose, other risk factors can be scored, even if they cannot perform the “Get‐Up‐and‐Go” test. If this test cannot be performed due to patients using a wheelchair or being bedridden, it may lead to an underestimation of the total fall risk assessment score.

For a diagnostic model with perfect classification, the AUC must be = 1; for a model with moderate to high accuracy, it should be >0.7; and for a model with low accuracy, the AUC is typically <0.5.^11^ As the cutoff score increases, sensitivity progressively decreases while specificity progressively increases; conversely, if the cutoff score decreases, sensitivity increases while specificity decreases.^28^ In Hendrich et al.'s (2020) study, HIIFRM scores ≥5 accounted for 36%, and in Ivziku et al.'s (2011) study, HIIFRM scores ≥5 accounted for 59.2%. In our study, HIIFRM scores ≥5 accounted for only 6% of the sample tested. Compared to previous studies, the number of cases classified as HIIFRM ≥5 in this study is significantly lower. This explains why a cutoff score of ≥2 instead of 5 shows greater accuracy in predicting falls in this study. Additionally, in Ivziku et al.'s (2011) study, focusing on cases classified as high risk with HIIFRM scores ≥5, among the 73 patients not classified as high risk, 2 older adults (2.7%) experienced falls. This indicates that while HIIFRM scores ≥5 is accurate in identifying a high‐risk population in some studies, it still misses some patients who are not classified as high risk but experience falls. In a study by Campanini et al. (2018) involving 147 elderly patients, an HIIFRM cutoff score of 5 yielded 100% sensitivity and 49% specificity, with an AUC of 0.779, indicating moderate predictive ability. Increasing the cutoff value to 8 reduced the sensitivity to 73% but increased the specificity to 72%. However, this resulted in the missing three fall patients being detected at the initial stage. Notably, the study had a small sample size.^28^ In this study, when the cutoff was set at ≥5, sensitivity and specificity were 29.5% and 86.3%, respectively, with an AUC of 0.57, indicating lower accuracy. Setting the cutoff at ≥2 increased sensitivity to 69.5% but decreased specificity to 64.4%, with an AUC of 0.745, indicating moderate accuracy for fall risk prediction. Lowering the cutoff to 2 identified an additional 101 fall events. Comparisons with Hendrich et al. (2020) suggest that reducing the cutoff point can detect more fall events. When the HIIFRM cutoff score was set at 5, only 6.0% of patients (*N* = 2843) were identified as high‐risk fall cases; however, when the cutoff score was lowered to 2, the percentage of identified high‐risk fall cases increased to 25.6% (*N* = 12,124), and an additional 101 fall events were identified. This is similar to the findings of Hendrich et al. (2020). Although the cutoff points are different, reducing the cutoff point can detect additional fall events.^3^

This study has some limitations. First, its retrospective nature makes it challenging to ascertain whether the assessment data provided by nurses accurately reflected patients' actual conditions at the time of evaluation. Additionally, there may be variations in HIIFRM scores among nurses. To control for this variation, all nurses at the study sites were required to complete a yearly standardized competency assessment regarding the definitions of HIIFRM risk factors and how to score fall risk. Second, hospitalized patients with underlying diseases receiving physiologically affecting medications and treatments may experience symptoms such as pain, fatigue, anxiety, sleep disturbances, transient ischemic attacks, arrhythmias, seizures, and impaired cognitive and physical functioning. This could potentially reduce the predictive ability of fall risk‐assessment tools. Third, when nurses use the HIIFRM to assess fall risk in hospitalized patients, they consider the patients' medication history, including the use of antiepileptic drugs and benzodiazepines. However, if patients failed to provide or disclose their medication information during the assessment, the scores for these items were recorded as 0, potentially leading to an inaccurate reflection of their current medication use. This limitation suggests that institutions utilizing this tool should incorporate essential factors such as sex, antiepileptic drug usage, and benzodiazepine usage directly into the assessment through an information system to prevent errors or omissions.

## 5 CONCLUSIONS

The psychometric characteristics of the HIIFRM can identify risk factors in hospitalized patients. This study indicates that the HIIFRM is a significant predictor of the recurrence of falls. For each additional point on the HIIFRM, the odds of a recurrence of a fall within 6 months increase by 1.23 times.^26^ Setting the cutoff score at ≥2 may increase the number of cases identified as high‐risk falls, but it can improve the detection of fall risk cases and facilitate timely implementation of fall prevention measures. In the Get‐Up‐and‐Go test, scoring should be based on the patient's actual condition. If the patient is comatose, they should be closely monitored, and a complete score should be assigned once the assessment is completed. If the patient is not comatose, other risk factors can be scored even if the patient cannot perform the Get‐Up‐and‐Go assessment. Based on the patient safety goal of “zero falls,” attention should be given to any potentially overlooked or neglected high‐risk fall cases. Monitoring fall risk among hospitalized patients is crucial for preventing falls in hospital settings.

## CONFLICT OF INTEREST STATEMENT

All authors declare no conflict of interest.
